# Early Predictors of Clinical Deterioration in a Cohort of 239 Patients Hospitalized for Covid-19 Infection in Lombardy, Italy

**DOI:** 10.3390/jcm9051548

**Published:** 2020-05-20

**Authors:** Maurizio Cecconi, Daniele Piovani, Enrico Brunetta, Alessio Aghemo, Massimiliano Greco, Michele Ciccarelli, Claudio Angelini, Antonio Voza, Paolo Omodei, Edoardo Vespa, Nicola Pugliese, Tommaso Lorenzo Parigi, Marco Folci, Silvio Danese, Stefanos Bonovas

**Affiliations:** 1 Department of Biomedical Sciences, Humanitas University, 20090 Pieve Emanuele, Milan, Italy; maurizio.cecconi@hunimed.eu (M.C.); enrico.brunetta@humanitas.it (E.B.); alessio.aghemo@hunimed.eu (A.A.); massimiliano.greco@hunimed.eu (M.G.); michele.ciccarelli@humanitas.it (M.C.); claudio.angelini@humanitas.it (C.A.); antonio.voza@humanitas.it (A.V.); paolo.omodei@humanitas.it (P.O.); edoardo.vespa@humanitas.it (E.V.); nicola.pugliese@humanitas.it (N.P.); tommaso.parigi@humanitas.it (T.L.P.); marco.folci@humanitas.it (M.F.); silvio.danese@hunimed.eu (S.D.); stefanos.bonovas@hunimed.eu (S.B.); 2 Humanitas Clinical and Research Center—IRCCS, 20089 Rozzano, Milan, Italy

**Keywords:** SARS-CoV-2, 2019 novel coronavirus, severe acute respiratory syndrome coronavirus 2, 2019-nCoV, COVID-19

## Abstract

We described features of hospitalized Covid-19 patients and identified predictors of clinical deterioration. We included patients consecutively admitted at Humanitas Research Hospital (Rozzano, Milan, Italy); retrospectively extracted demographic; clinical; laboratory and imaging findings at admission; used survival methods to identify factors associated with clinical deterioration (defined as intensive care unit (ICU) transfer or death), and developed a prognostic index. Overall; we analyzed 239 patients (29.3% females) with a mean age of 63.9 (standard deviation [SD]; 14.0) years. Clinical deterioration occurred in 70 patients (29.3%), including 41 (17.2%) ICU transfers and 36 (15.1%) deaths. The most common symptoms and signs at admission were cough (77.8%) and elevated respiratory rate (34.1%), while 66.5% of patients had at least one coexisting medical condition. Imaging frequently revealed ground-glass opacity (68.9%) and consolidation (23.8%). Age; increased respiratory rate; abnormal blood gas parameters and imaging findings; coexisting coronary heart disease; leukocytosis; lymphocytopenia; and several laboratory parameters (elevated procalcitonin; interleukin-6; serum ferritin; C-reactive protein; aspartate aminotransferase; lactate dehydrogenase; creatinine; fibrinogen; troponin-I; and D-dimer) were significant predictors of clinical deterioration. We suggested a prognostic index to assist risk-stratification (C-statistic; 0.845; 95% CI; 0.802–0.887). These results could aid early identification and management of patients at risk, who should therefore receive additional monitoring and aggressive supportive care.

## 1. Introduction

In late December 2019, clusters of patients with pneumonia of an unknown cause were reported in Wuhan, Hubei Province in China. Subsequently, a novel coronavirus (SARS-CoV-2), causing a severe acute respiratory syndrome, had been isolated from those patients [1,2,3]. In recognition of its global transmission, the World Health Organization declared Covid-19 as a pandemic on 11 March 2020 [4]. On 21 February, the first case of Covid-19 was detected in Italy. As of 23 April 2020, the Italian National Healthcare Service has declared about 190,000 cases of Covid-19 with 25,549 deaths.

Covid-19 has a spectrum of manifestations ranging from asymptomatic infection, to mild upper respiratory symptoms, to bilateral pneumonia with respiratory failure requiring advanced respiratory support [5,6,7,8]. The rapid diffusion rate of the disease in the population, due to asymptomatic carriers, associated with the possible sudden deterioration of clinical conditions requiring critical care admission has contributed to the exceeding of hospital and intensive care units (ICUs) capacity in several Italian hospitals [9,10,11,12]. To increase capacity, elective surgeries were cancelled, semi-elective procedures postponed, and operating rooms turned into makeshift ICUs [9,10,12,13].

Since the current Covid-19 pandemic poses a major strain especially on critical care facilities [9,10,11,12], being able to stratify patient risk at admission would help focus treatment efforts to potentially prevent deterioration [14]. Although recent studies investigating predictors of poor prognosis at an early stage identified potential risk factors including older age, high SOFA score, d-dimer, lymphopenia, and the presence of secondary infection or cardiac disease [8,15], such evidence has not been reported from studies conducted outside of China yet.

In this paper, we described the clinical characteristics and outcomes of a cohort of 239 SARS-CoV-2 positive patients admitted to Humanitas Research Hospital (Milan, Italy) and assessed predictors of clinical deterioration, defined as ICU transfer or death. This study could have significant implications in the clinical management of Covid-19 patients.

## 2. Materials and Methods

### 2.1. Study Design and Participants

This retrospective cohort study included all males and non-pregnant females, 18 years of age or older, consecutively admitted to Humanitas Research Hospital between 22 February and 22 March, 2020, with a laboratory-confirmed diagnosis of Covid-19.

Hospital admission criteria were based on a positive assay for SARS-CoV-2 associated with respiratory failure requiring oxygen therapy, or radiological evidence of significant pulmonary infiltrates on a chest computed tomography (CT) scan, or reduction in respiratory/cardiopulmonary reserve as assessed by a 6 min walking test, or due to frailty related with patient comorbidity.

We assessed a composite outcome of ICU transfer or death. Patients transferred to ICU were those requiring invasive ventilation or non-invasive mechanical ventilation with an oxygen fraction over 60%. Patients with continuous positive airway pressure therapy (CPAP) were followed up by an ICU outreach team and ward physicians in Covid-19 wards. Acute respiratory syndrome (ARDS) was defined according to the Berlin definition [16]. Acute kidney injury (AKI) was diagnosed according to the Kidney Disease Improving Global Outcomes (KDIGO) clinical practice guideline [17].

### 2.2. Laboratory Test, Demographic, and Medical History

Laboratory testing at hospital admission included: complete blood count, renal and liver function (transaminase, total/direct/indirect bilirubin, gamma-glutamyl transferase, alkaline phosphatase), creatinine kinase, lactate dehydrogenase, myocardial enzymes, electrolytes and triglycerides. A panel of acute phase reactant including interleukin-6 (IL-6), serum ferritin, d-dimer, c-reactive protein, fibrinogen procalcitonin was performed routinely. Body temperature, blood pressure, heart rate, peripheral saturation and respiratory rate were measured in all patients. Chest CT scan and arterial blood gas analysis were performed in the emergency department.

Pneumococcal and Legionella urinary antigen test were routinely performed at hospital admission. Nasopharyngeal swab for influenza A, B, H1N1 was routinely performed to exclude co-infection. Additional microbiological tests were performed (bacterial cultures of sputum, blood and urine) when suggested by clinical conditions. We obtained a comprehensive medical history from patients.

Positivity was assessed with reverse-transcriptase–polymerase-chain-reaction (RT-PCR) assay for SARS-CoV-2 on a respiratory tract sample tested by our laboratory as of 16 March 2020, in accordance with the protocol established by the World Health Organization (WHO; Geneva, Switzerland) [18]. Previously, respiratory specimens were tested at San Matteo Hospital (Pavia, Italy). Due to the high false-negative rate of RT-PCR from a pharyngeal swab, two different swabs were performed in each patient to increase the detection rate [19]. In cases of a negative assay, but suggestive clinical manifestations, presence of contact history or suggestive radiological evidence for Covid-19, the detection was performed on bronchoalveolar lavage fluid or endotracheal aspirate, which has higher diagnostic accuracy.

Demographic, clinical, laboratory, and outcome data were obtained from electronic medical records and were checked by three expert physicians. For a few clinical variables and in the case of a high proportion of missing information, the same physicians manually reviewed the patients’ chart notes using a standardized data collection form (Table S1, Supplementary Appendix). The data cut-off was 25 March 2020. The study was approved by the local Ethical Committee, and the requirement for informed consent was waived.

### 2.3. Statistical Methods

Descriptive statistics included means with standard deviations (SD) and medians with interquartile ranges (IQR) for continuous variables, and frequency analyses (percentages) for categorical variables.

To identify risk factors associated with clinical deterioration, leading to ICU transfer or death in hospitalized Covid-19 patients, we used time-to-event (survival) methods for censored observations. The composite study endpoint was “ICU transfer or death” within 20 days from hospital admission. Time to event was defined as the time from hospital admission until the date of event or censoring. Patients discharged early and alive from the hospital, and without having experienced ICU transfer, were considered event-free through day 20 [20]. Kaplan–Meier estimates were used to draw the cumulative incidence curves, compared by log-rank tests, as well as by univariable and multivariable Cox proportional hazards (PH) models of relevant prognostic factors. The analyses were based on non-missing data (missing data not imputed). We followed a standard approach for model selection.

In the univariable Cox PH analysis, a criterion of *p* ≤ 0.10 was used to identify candidate predictors. Additionally, variables were selected according to a review of the literature and consensus opinion by an expert group of physicians and methodologists. Then, we fitted the multivariable model and used backwards selection procedure to eliminate those variables not significant in the multivariable framework. The criterion of *p* ≤ 0.05 was used for determining which ones to eliminate. After fitting the model, the PH assumption was examined on the basis of Schoenfeld residuals. The hazard ratios (HR) were presented with their 95% confidence intervals (CI) and the respective *p*-values.

We developed a preliminary prognostic index for clinical deterioration (i.e., ICU transfer or death) of hospitalized Covid-19 patients by converting the beta coefficients from the multivariable model to integer values, while preserving monotonicity and simplicity. We computed the Harrell’s C statistic with its 95% CI to measure the predictive power of our prognostic index. To allow for an easy risk stratification, we divided our score into three groups corresponding to low, intermediate, and high risk, and reported the corresponding observed risk of “ICU transfer or death”. Calibration was tested by plotting predicted versus observed Kaplan–Meier curves across the three groups of risk [21,22,23,24,25].

Stata 15.0 software was used to analyze the data (Stata Corp., College Station, TX, USA). *P*-values less than 0.05 were considered statistically significant. All tests were two-sided. No adjustment for multiplicity was applied.

## 3. Results

From 22 February to 22 March 2020, 239 patients had been admitted to Humanitas Research Hospital with confirmed Covid-19. They were 169 males (70.7%) and 70 females (29.3%); their mean age was 63.9 ± 14.0 years. The most common symptom at admission was a cough (77.8%). Physical examination revealed increased respiratory rates (≥ 20 breaths per minute; 34.1%) and body temperature (median, 37.3; IQR, 36.5–38.0 °C).

Some patients showed abnormal blood gas analysis findings (decreased partial pressure of oxygen (PaO_2_, 19.0%) and elevated partial pressure of carbon dioxide (PaCO_2_, 10.0%)). About one third of the patients showed signs of moderate (21.2%) or severe (11.1%) acute respiratory distress syndrome.

Among the overall population, 66.5% had at least one coexisting medical condition. Hypertension (50.2%), diabetes type 2 (21.8%), coronary heart disease (CHD, 16.7%), atrial fibrillation (11.3%), active neoplasia (9.6%), chronic obstructive pulmonary disease (9.2%), and chronic kidney disease (8.4%) were the most common comorbidities. The majority (92.8%) of the CT scans performed at the time of admission revealed abnormal results (ground-glass opacity (68.9%) and consolidation (23.8%)).

Lymphocytopenia was present in 50.6% of the patients, thrombocytopenia in 26.5%, leukocytosis in 20.1% and leukopenia in 13.0%. Most patients had high levels of lactate dehydrogenase (74.7%), D-dimer (62.3%) and C-reactive protein (64.9%). Less common were elevated levels of creatine kinase (37.7%), creatinine (31.8%), aspartate aminotransferase (28.5%), troponin (27.7%) and alanine aminotransferase (9.4%). Demographic, clinical, and laboratory characteristics of the patients are shown in Table 1.

**Figure 1:**
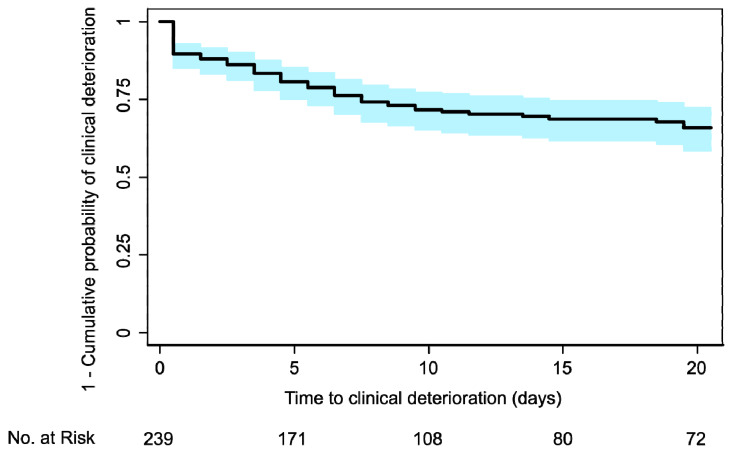
Kaplan–Meier curve in patients with confirmed COVID-19 admitted to Humanitas Research Hospital, between 22 February to 22 March 2020. The composite endpoint was ICU transfer or death. The overall 20-day event-free survival was 0.66 (95% CI, 0.59–0.72). CI: confidence interval; ICU: intensive care unit.

The Kaplan–Meier curve showed an overall 20-day event-free survival (determined as “1–cumulative probability of clinical deterioration”) of 0.66 (95% CI, 0.59–0.72) (Figure 1). Most patients received a combination of hydroxychloroquine (87.4%) and antiviral therapies (darunavir/cobicistat (47.3%), lopinavir/ritonavir (40.6%)), while many received empirical antibiotics (ceftriaxone (74.1%), azithromycin (31.4%), piperacillin/tazobactam (14.2%)), tocilizumab (3.3%), and steroids (5.6%).

As of 25 March 2020, 71 patients (21.8%) had been discharged, 132 (55.2%) were still hospitalized, while the composite endpoint of clinical deterioration occurred in 70 patients (29.3%), including 41 (17.2%) who were admitted to the ICU, and 36 (15.1%) who died. Of the 41 patients admitted to the ICU, 31 were still hospitalized, 3 had been discharged, and 7 had died.

Several clinical and laboratory characteristics at admission were associated with clinical deterioration (Table 2). Advanced age, increased respiratory rate, a low PaO_2_, a high PaCO_2_, an elevated ratio of PaO_2_ to FiO_2_ (fraction of inspired oxygen), coexisting CHD, abnormal imaging features in the CT scan, leukocytosis (white blood cell count > 10 × 10^9^/L), lymphocytopenia (lymphocyte count < 1 × 10^9^/L), and elevated levels of certain laboratory parameters (i.e., procalcitonin, interleukin-6, serum ferritin, C-reactive protein, aspartate aminotransferase, serum creatinine, lactate dehydrogenase, fibrinogen, troponin-I, and D-dimer) were statistically significant predictors of clinical deterioration leading to ICU transfer or death in hospitalized Covid-19 patients. Gender, body temperature, body mass index (BMI), and clinical symptoms at admission were not associated with the risk of clinical deterioration (Table 2 and Figure S1, Supplementary Appendix).

On multivariable analysis, the factors predictive of clinical deterioration were respiratory rate (adjusted HR (aHR) for ≥20 vs. <20 breaths per minute, 1.89; 95% CI, 1.11–3.23; *p* = 0.020), an elevated ratio of PaO_2_ to FiO_2_ (aHR for 200 < PaO_2_/FiO_2_ ≤ 300 vs. PaO_2_/FiO_2_ > 300, 1.96; 95% CI, 0.82–4.67; *p* = 0.13; aHR for 100 < PaO_2_/FiO_2_ ≤ 200 vs. < PaO_2_/FiO_2_ > 300, 5.16; 95% CI, 2.34–11.4; *p* < 0.001; and aHR for 100 ≤ PaO_2_/FiO_2_ vs PaO_2_/FiO_2_ > 300, 7.29; 95% CI, 3.05–17.4; *p* < 0.001), coexisting CHD (aHR, 2.02; 95% CI, 1.13–3.64; *p* = 0.018), C-reactive protein (aHR per 1 mg/dL increase, 1.06; 95% CI, 1.03–1.10; *p* < 0.001), and serum creatinine (aHR for ≥ 1.10 vs. < 1.10 mg/dL, 2.00; 95% CI, 1.17–3.41; *p* = 0.011) (Table 2). There was no evidence that the PH assumption was violated (*p* = 0.376). 

### Prognostic Index

We developed a preliminary tool to predict clinical deterioration. We provided easy-to-apply instructions to derive the score for each patient given his/her respiratory rate, ratio of PaO_2_ to FiO_2_, medical history of CHD, C-reactive protein and serum creatinine levels (Figure S2, Supplementary Appendix).

The prognostic index showed high predictive accuracy (Harrell’s C, 0.845; 95% CI, 0.802–0.887). As a sensitivity analysis, we reassessed the predictive power of this tool after exclusion of patients who died or were transferred to the ICU within the first day of hospitalization. The prognostic index was robust (Harrell’s C, 0.812; 95% CI, 0.761–0.870).

**Figure 2:**
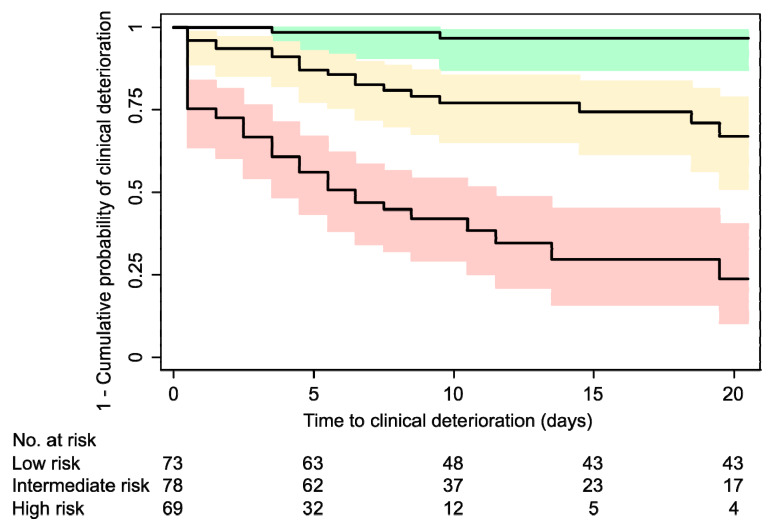
Kaplan–Meier curves by risk-category. The composite endpoint was ICU transfer or death. The 20-day event-free survival was 0.97 (95% CI, 0.87–0.99) for the “low risk” group (upper curve), 0.67 (95% CI, 0.51–0.79) for the “intermediate risk” (intermediate), and 0.24 (95% CI, 0.10–0.40) for the “high risk” group (lower curve). CI: confidence interval; ICU: intensive care unit.

We divided our score into three risk-categories corresponding to “low-risk” (score ≤ 15), “intermediate-risk” (15 < score < 40), and “high-risk” for clinical deterioration (score ≥ 40). The corresponding observed 20-day event-free survival was 0.97 (95% CI, 0.87–0.99) for the “low-risk” category, 0.67 (95% CI, 0.51–0.79) for the “intermediate-risk” category, and 0.24 (95% CI, 0.10–0.40) for the “high-risk” category (Table 3 and Figure 2). There was optimal agreement between the predicted and observed survival curves across the three risk strata (Figure S3, Supplementary Appendix).

## 4. Discussion

The emerging Covid-19 pandemic has posed serious strain on Italian hospitals, and, in certain areas, has severely challenged the capacity to provide adequate care to all patients [9,10,12]. Besides supportive care, no effective medications have been identified to date. Although close follow-up is sufficient to manage most non-severe cases, aggressive treatments requiring hospital admission and intensive care are needed in more than 20% of cases [5,7,26]. As ICU bed availability ranges from 4 to 20 per 100,000 population in most western countries [27], even just 1% of patients requiring ICU transfer could easily overwhelm hospital surge capacity. In this scenario, rationalizing the available healthcare resources is of paramount importance to guarantee adequate care to the highest number of patients [11,12]. In this study, we reported the clinical characteristics and outcomes of a cohort of 239 patients with confirmed Covid-19 infection at the Humanitas Research Hospital, and identified predictors of clinical deterioration at admission, with the aim to assist risk-stratification of newly admitted patients.

The mean age of our patients was 5–10 years higher than what was reported in previous studies conducted in China [7,8]. This factor alone probably justifies the higher mortality reported in this Italian cohort (15.2%), as age has been associated with mortality [7,8]. Whilst Covid-19 has affected more males than females with a ratio of approximately 2:1, as confirmed in other studies [1,5,7,8], gender was not associated with clinical deterioration. Similar considerations apply to BMI, which was in the range of overweight in most Covid-19 in-hospital patients. 

The oxygenation index PaO_2_/FiO_2_ was independently associated with risk of clinical deterioration. Patients with PaO_2_/FiO_2_ < 100 were 12 times more likely to deteriorate than patients with values > 300. PaO_2_/FiO_2_ is the most widely used among oxygenation indexes, and is included in both the acute respiratory syndrome definition and in sepsis management guidelines [16,28]. Although PaO_2_/FiO_2_ ratio has several limitations [29,30], it has been independently associated with mortality in the other cohort of acute respiratory failures [31]. PaO_2_/FiO_2_ and other oxygenation indexes are included in the most used indices for mortality prediction, Sepsis-related Organ Failure Assessment (SOFA) and Acute Physiology and Chronic Health Disease Classification System II (APACHE II) score [32,33]. Respiratory rate is one of the simplest clinical indexes, and is also included in APACHE II and in Q-SOFA (quick-SOFA) score [33,34]. In Covid-19 patients, a respiratory rate over 24 breaths per min has been previously associated with mortality [35], in agreement with our findings.

Pneumonia and septic shock have been closely associated in other populations [36]. This is not confirmed in Covid-19 patients according to our findings and previous published data. Guan et al. [37] reported an incidence of septic shock as low as 1% in Covid-19 patients. This was consistent with our data, yielding no association between hemodynamic data at admission and worsening of clinical conditions.

Coronary heart disease, respiratory rate, lymphocyte count, lactate dehydrogenase, creatinine, creatine kinase, troponin I, D-dimer, ferritin, IL-6 and procalcitonin were associated with the risk of clinical deterioration, similarly to what was found by Zhou et al. [8]. Coronary heart disease was a strong predictor of deterioration in our cohort, and has been associated with poor clinical outcomes in other respiratory viral infections [38]. Troponin I was elevated in about one fourth of patients compared to half patients in other reports, and was a strong predictor of ICU transfer or death [8]. Most studies describing critically ill patients very frequently reported an increase in troponin levels without a clear association with myocardial dysfunction or myocarditis in ICU patients [39]. The increase of troponin is not clear evidence of myocarditis or myocardial infarction; it could be related to a direct viral damage of myocardium (through ACE2 expressed by myocardiocytes), a hyperinflammatory syndrome, cardiac microvascular damage or hypoxia-induced myocardial injury [38]. A slight increase of troponin levels can also be attributed to the high frequency of acute kidney injury in Covid-19 patients. 

A rise in serum creatinine doubled the hazard for deterioration in our population. Serum creatine at admission is a marker for AKI, which is strongly associated with mortality in hospitalized patients [40]. Zhou et al. [8] reported a 50-fold higher frequency of AKI among non-survivors. The etiology of Covid-19 related AKI is yet to be determined, but we hypothesize that it could be due to dehydration after several days of high fever and diarrhea, to a high level of positive end expiratory pressure, multiorgan failure, or direct viral cytopathic injury of renal cells.

Imaging features obtained through a chest CT scan were strong predictors of outcome, however we decided not to include them in our model because this diagnostic tool may not be available in all countries or hospitals. Accordingly, several societies including the Royal College of Radiology currently report no role for the CT scan in Covid-19 diagnosis [41]. In our multivariable model, inclusion of chest CT scan added a negligible increase in the tool’s predictive power.

Our study has limitations. First, since the data were retrospectively collected, we could not assess all laboratory parameters in all patients, including IL-6. Second, we considered eligible all consecutively admitted Covid-19 patients, irrespective of the latency since symptom onset. As a consequence, we also included patients who required ICU transfer very early after admission. We acknowledge that these patients would not benefit from a risk-stratification at admission. However, we were able to substantially verify the validity of our risk-stratification tool after excluding these severely ill patients. Third, we conducted multiple comparisons, and this could increase type I error, thus our analyses should be interpreted with due caution. Finally, our prognostic index possibly needs to be improved by external prospective cohorts, to account for potential differences in healthcare systems, measurement methods, definitions of predictors, and subject characteristics or context. 

Our study has several strengths. First, we had at our disposal a database rich in clinical, laboratory and imaging findings, including CT, in more than two hundred patients with few missing data, which may be difficult to find in other settings. We applied appropriate time-to-event methodology and developed a simple and easy-to-apply clinical scoring system; its C-index of 0.845 (0.802–0.887) demonstrates excellent predictive power.

One of the causes of the Italian Covid-19 crisis, is probably the initial massive admission to hospitals of patients with low risk of severe deterioration, coupled with many hospitals being unprepared to identify and contain such a massive flux of patients requiring respiratory support. Our study provides novel information about Covid-19 and its clinical outcomes. There is an urgent need to guide patient management and focus treatment efforts, especially in conditions where the system is overwhelmed. We believe that our results could aid early identification and management of patients at risk of clinical deterioration, who should, therefore, receive additional monitoring and aggressive supportive care.

## Figures and Tables

**Table 1:** Demographic, clinical, and laboratory characteristics of hospitalized Covid-19 patients (*n* = 239).

	Mean ± SD; Median (IQR) or *n* (%)
**Demographic Characteristics**	
Age (years; *n* = 239)	63.9 ± 14.0; 65.2 (53.8–74.5)
≥65	120 (50.2)
<65	119 (49.8)
Gender (*n* = 239)	
Female	70 (29.3)
Male	169 (70.7)
**Clinical Characteristics**	
**Signs and Symptoms at Admission**	
Systolic blood pressure (mmHg; *n* = 238)	125 ± 16.8; 125 (115–135)
Pulse (beats per minute; *n* = 238)	82 ± 14; 81 (71–90)
Respiratory rate (breaths per minute; *n* = 232)	19.3 ± 3.2; 18 (18–20)
Temperature (°C; *n* = 239)	37.3 ± 0.97; 37.3 (36.5–38.0)
Body mass index (N=194)	27.1 ± 4.76; 26.3 (24.0–29.4)
Cough (*n* = 216)	168 (77.8)
Myalgia (*n* = 176)	22 (12.5)
PaO_2_ (mmHg; *n* = 226)	73.9 ± 20.0; 73.0 (63.0–80.0)
PaCO_2_ (mmHg; *n* = 201)	36.0 ± 8.30; 35.0 (31.0–39.0)
Ratio of PaO_2_ to FiO_2_ (*n* = 226)	265 ± 121; 282 (161–357)
**Comorbid Conditions**	
Hypertension (*n* = 239)	120 (50.2)
Diabetes type 2 (*n* = 239)	52 (21.8)
Coronary heart disease (*n* = 239)	40 (16.7)
Atrial fibrillation (*n* = 239)	27 (11.3)
Active neoplasia (*n* = 239)	23 (9.6)
Chronic obstructive pulmonary disease (*n* = 239)	22 (9.2)
Chronic kidney disease (*n* = 238)	20 (8.4)
Other (*n* = 239)	26 (10.9)
Comorbidities (*n* = 239)	
0	80 (33.5)
1	70 (29.3)
≥2	89 (37.2)
**Imaging Features** (*n* = 235)	
Normal/non-specific signs	17 (7.2)
Ground-glass opacity	162 (68.9)
Consolidation	56 (23.8)
**Laboratory Characteristics**	
**Blood Routine**	
White blood cells (×10^9^/L; normal range 4.0–10.0; *n* = 238)	7.70 ± 5.25; 6.34 (4.93–9.39)
Lymphocytes (×10^9^/L; normal range 1.0–4.0; *n* = 237)	1.06 ± 0.73; 0.90 (0.70–1.20)
Platelets (×10^9^/L; normal range 150–400; *n* = 238)	206 ± 85.7; 195 (148–240)
Hemoglobin (g/dL; normal range 13.0–16.0; *n* = 239)	13.9 ± 1.88; 14.0 (12.7–15.2)
**Infection-Related Biomarkers**	
Procalcitonin (ng/mL; normal range 0.05–0.5; *n* = 222)	0.62 ± 1.62; 0.16 (0.08–0.38)
Interleukin-6 (pg/mL; normal range < 6.4; *n* = 114)	73.4 ± 98.6; 43.5 (16.0–89.0)
Serum ferritin (ng/mL; normal range 23.9–336.2; *n* = 212)	765 ± 858; 498 (228–933)
C-reactive protein (mg/dL; normal range < 1.0; *n* = 239)	9.70 ± 8.19; 7.73 (2.69–14.7)
**Blood Biochemistry**	
Alanine aminotransferase (U/L; normal range < 51; *n* = 239)	36.5 ± 37.6; 27.0 (17.0–42.0)
Aspartate aminotransferase (U/L; normal range < 51; *n* = 239)	46.2 ± 39.9; 35.0 (25.0–54.0)
Gamma-glutamyl transpeptidase (U/L; normal range < 55; *n* = 218)	61.9 ± 79.5; 41.0 (22.0–69.0)
Alkaline phosphatase (U/L; normal range 40–150; *n* = 214)	87.5 ± 49.1; 74.0 (59.0–93.0)
Total bilirubin (mg/dL; normal range 0.3–1.2; *n* = 239)	0.80 ± 1.06; 0.60 (0.50–0.80)
Direct bilirubin (mg/dL; normal range < 0.3; *n* = 229)	0.26 ± 0.63; 0.20 (0.10–0.30)
Indirect bilirubin (mg/dL; normal range 0.05–1.10; *n* = 218)	0.60 ± 0.42; 0.50 (0.40–0.70)
Serum creatinine (mg/dL; normal range 0.67–1.17; *n* = 239)	1.11 ± 0.74; 0.93 (0.75–1.17)
Creatine kinase (U/L; normal range < 172; *n* = 239)	271 ± 629; 120 (68.0–226)
Lactate dehydrogenase (U/L; normal range < 248; *n* = 234)	363 ± 177; 316 (243–422)
Triglycerides (mg/dL; normal range 10–150; *n* = 210)	143 ± 108; 120 (92.0–157)
**Coagulation Function and Other Biomarkers**	
Fibrinogen (mg/dL; normal range 160–400; *n* = 229)	570 ± 162; 556 (450–665)
D-dimer (μg/mL; normal range 0.2–0.35; *n* = 212)	1.56 ± 4.27; 0.47 (0.29–0.89)
Troponin-I (ng/L; normal range 1–19.8; *n* = 206)	128 ± 1242; 8.10 (3.20–22.0)

FiO_2_: fraction of inspired oxygen; IQR: interquartile range; PaCO_2_: partial pressure of carbon dioxide; PaO_2_: partial pressure of oxygen; SD: standard deviation.

**Table 2:** Predictors of Clinical Deterioration Leading to “ICU Transfer or Death” in Hospitalized COVID-19 Patients: Results of Time-to-Event Analysis.

	Log-Rank Test		Univariable Cox PH Model			Multivariable Cox PH Model		
Baseline Parameter	Chi-Squared (d.f.)	*p*-Value	HR	(95% CI)	*p*-Value	Adjusted HR	(95% CI)	*p*-Value
**Demographic characteristics**								
Age (per 10-year increase)	―	―	1.23	(1.03–1.45)	0.020			
Age (≥65 vs. <65 years)	3.63 (1)	0.057	1.57	(0.97–2.54)	0.065			
Gender (male vs. female)	1.92 (1)	0.16	1.47	(0.84–2.56)	0.18			
**Clinical Characteristics**								
**Signs and Symptoms at Admission**								
Systolic blood pressure (per 10 mmHg increase)	―	―	1.06	(0.92–1.22)	0.43			
Systolic blood pressure (≥140 vs. <140 mmHg)	0.01 (1)	0.92	1.03	(0.58–1.82)	0.93			
Pulse (per 10 beats per minute increase)	―	―	1.16	(0.98–1.37)	0.085			
Pulse (≥100 vs. <100 beats per minute)	3.09 (1)	0.079	1.71	(0.92–3.19)	0.090			
Respiratory rate (per 1 breath per minute increase)	―	―	1.15	(1.09–1.21)	<0.001			
Respiratory rate (≥20 vs. <20 breaths per minute)	18.9 (1)	<0.001	2.79	(1.71–4.58)	<0.001	1.89	(1.11–3.23)	0.020
Temperature (per 1 °C increase)	―	―	0.87	(0.68–1.12)	0.28			
Temperature (≥37.3 vs. <37.3 °C)	0.12 (1)	0.73	0.92	(0.58–1.47)	0.74			
Body mass index (per 1 kg/m^2^ increase)	―	―	1.01	(0.96–1.06)	0.61			
Body mass index								
<25	0.07 (2)	0.97	1.00	(reference)				
25–30			0.97	(0.55–1.73)	0.93			
>30			0.91	(0.45–1.84)	0.80			
Cough	0.04 (1)	0.85	0.95	(0.53–1.69)	0.85			
Myalgia	1.55 (1)	0.21	0.54	(0.19–1.49)	0.23			
PaO_2_ (per 10 mm Hg increase)	―	―	0.94	(0.82–1.08)	0.41			
PaO_2_ (≥60 vs. <60 mm Hg)	8.22 (1)	0.004	0.48	(0.28–0.81)	0.006			
PaCO_2_ (per 1 mmHg increase)	―	―	1.08	(1.06–1.11)	<0.001			
PaCO_2_ (≥45 vs. <45 mm Hg)	111 (1)	<0.001	15.2	(7.58–30.4)	<0.001			
Ratio of PaO_2_ to FiO_2_ (per 10 units increase)	―	―	0.92	(0.90–0.94)	<0.001			
PaO_2_/FiO_2_ > 300	74.0 (3)	<0.001	1.00	(reference)		1.00	(reference)	
200 < PaO_2_/FiO_2_ ≤ 300			3.37	(1.49–7.61)	0.003	1.96	(0.82–4.67)	0.129
100 < PaO_2_/FiO_2_ ≤ 200			10.6	(5.09–22.2)	<0.001	5.16	(2.34–11.4)	<0.001
100 ≤ PaO_2_/FiO_2_			12.3	(5.43–27.9)	<0.001	7.29	(3.05–17.4)	<0.001
**Comorbid Conditions**								
Hypertension	2.69 (1)	0.10	1.47	(0.91–2.37)	0.11			
Diabetes type 2	1.02 (1)	0.31	1.30	(0.77–2.21)	0.33			
Coronary heart disease	9.23 (1)	0.002	2.15	(1.28–3.62)	0.004	2.02	(1.13–3.64)	0.018
Atrial fibrillation	1.25 (1)	0.26	1.43	(0.75–2.72)	0.28			
Active neoplasia	0.18 (1)	0.68	1.17	(0.56–2.44)	0.68			
Chronic obstructive pulmonary disease	1.05 (1)	0.30	1.43	(0.71–2.87)	0.32			
Chronic kidney disease	2.75 (1)	0.097	1.77	(0.88–3.57)	0.11			
Any comorbid condition	5.99 (1)	0.014	1.98	(1.12–3.51)	0.019			
**Imaging Features**								
Normal/non-specific signs	33.9 (2)	<0.001	1.00	(reference)				
Ground-glass opacity			1.33	(0.41–4.34)	0.63			
Consolidation			4.67	(1.43–15.3)	0.011			
**Laboratory characteristics**								
**Blood Routine**								
White blood cells (per 1 × 10^9^/L increase)	―	―	1.05	(1.02–1.08)	<0.001			
White blood cells (×10^9^/L)								
<4	5.69 (2)	0.058	1.19	(0.58–2.45)	0.64			
4–10			1.00	(reference)				
>10			1.85	(1.09–3.15)	0.022			
Lymphocytes (per 1 × 10^9^/L increase)	―	―	0.60	(0.35–1.01)	0.055			
Lymphocytes (≥1 vs. <1 × 10^9^/L)	5.42 (1)	0.020	0.57	(0.35–0.93)	0.025			
Platelets (per 100 × 10^9^/L increase)	―	―	1.26	(0.97–1.65)	0.088			
Platelets (≥150 vs. <150 × 10^9^/L)	0.35 (1)	0.56	0.86	(0.50–1.46)	0.57			
Hemoglobin (per 1 g/dL increase)	―	―	0.83	(0.74–0.93)	0.002			
Hemoglobin (≥13 vs. <13 g/dL)	3.33 (1)	0.068	0.65	(0.40–1.05)	0.077			
**Infection-Related Biomarkers**								
Procalcitonin (per 1 ng/mL increase)	―	―	1.11	(1.02–1.21)	0.016			
Procalcitonin (≥0.5 vs. <0.5 ng/mL)	20.2 (1)	<0.001	2.86	(1.74–4.69)	<0.001			
Interleukin-6 (per 100 pg/mL increase)	―	―	1.31	(1.00–1.73)	0.049			
Interleukin-6 (≥100 vs. <100 pg/mL)	8.81 (1)	0.003	4.34	(1.50–12.6)	0.007			
Serum ferritin (per 100 ng/mL increase)	―	―	1.03	(1.01–1.06)	0.004			
Serum ferritin (≥336.2 vs. <336.2 ng/mL)	6.96 (1)	0.008	2.49	(1.23–5.04)	0.012			
C-reactive protein (per 1 mg/dL increase)	―	―	1.09	(1.06–1.11)	<0.001	1.06	(1.03–1.10)	<0.001
C-reactive protein (≥5 vs. <5 mg/dL)	18.4 (1)	<0.001	3.63	(1.90–6.92)	<0.001			
**Blood Biochemistry**								
Alanine aminotransferase (per 10 U/L increase)	―	―	1.03	(0.98–1.08)	0.24			
Alanine aminotransferase (≥50 vs. <50 U/L)	1.52 (1)	0.22	1.41	(0.80–2.46)	0.23			
Aspartate aminotransferase (per 10 U/L increase)	―	―	1.05	(1.01–1.09)	0.010			
Aspartate aminotransferase (≥50 vs. <50 U/L)	6.62 (1)	0.010	1.85	(1.13–3.01)	0.014			
Gamma-glutamyl transpeptidase (per 100 U/L increase)	―	―	0.96	(0.66–1.41)	0.85			
Gamma-glutamyl transpeptidase (≥55 vs. <55 U/L)	1.03 (1)	0.31	1.33	(0.76–2.34)	0.32			
Alkaline phosphatase (per 100 U/L increase)	―	―	0.92	(0.50–1.69)	0.79			
Alkaline phosphatase (≥150 vs. <150 U/L)	0.57 (1)	0.45	0.64	(0.20–2.07)	0.46			
Total bilirubin (per 1 mg/dL increase)	―	―	1.03	(0.86–1.23)	0.73			
Total bilirubin (≥1.2 vs. <1.2 mg/dL)	2.85 (1)	0.091	1.68	(0.90–3.12)	0.103			
Direct bilirubin (per 1 mg/dL increase)	―	―	1.06	(0.80–1.41)	0.67			
Direct bilirubin (≥0.3 vs. <0.3 mg/dL)	8.69 (1)	0.003	2.00	(1.23–3.24)	0.005			
Indirect bilirubin (per 1 mg/dL increase)	―	―	0.27	(0.07–0.98)	0.047			
Indirect bilirubin (≥1.1 vs. <1.1 mg/dL)	0.06 (1)	0.81	0.87	(0.27–2.78)	0.82			
Serum creatinine (per 1 mg/dL increase)	―	―	1.39	(1.15–1.69)	0.001			
Serum creatinine (≥1.10 vs. <1.10 mg/dL)	17.8 (1)	<0.001	2.60	(1.62–4.17)	<0.001	2.00	(1.17–3.41)	0.011
Creatine kinase (per 100 U/L increase)	―	―	1.01	(0.98–1.04)	0.61			
Creatine kinase (≥172 vs. <172 U/L)	4.21 (1)	0.040	1.61	(1.01–2.58)	0.047			
Lactate dehydrogenase (per 100 U/L increase)	―	―	1.39	(1.27–1.52)	<0.001			
Lactate dehydrogenase (≥250 vs. <250 U/L)	8.12 (1)	0.004	2.62	(1.30–5.33)	0.007			
Triglycerides (per 100 mg/dL increase)	―	―	1.11	(0.96–1.29)	0.17			
Triglycerides (≥150 vs. <150 mg/dL)	2.93 (1)	0.087	1.69	(0.92–3.12)	0.093			
**Coagulation Function and Other Biomarkers**								
Fibrinogen (per 100 mg/dL increase)	―	―	1.32	(1.14–1.52)	<0.001			
Fibrinogen (≥400 vs. <400 mg/dL)	1.19 (1)	0.28	1.53	(0.70–3.35)	0.29			
D-dimer (per 1 μg/L increase)	―	―	1.06	(1.03–1.09)	<0.001			
D-dimer (≥0.35 vs. <0.35 μg/L)	17.9 (1)	<0.001	3.58	(1.87–6.86)	<0.001			
Troponin-I (per 1000 ng/L increase)	―	―	1.15	(1.03–1.27)	0.010			
Troponin-I (≥20 vs. <20 ng/L)	23.0 (1)	<0.001	3.08	(1.87–5.08)	<0.001			

FiO_2_: fraction of inspired oxygen; HR: hazard ratio; PaCO_2_: partial pressure of carbon dioxide; PaO_2_: partial pressure of oxygen; PH: proportional hazard.

**Table 3:** Risk stratification: observed risk of “intensive care unit (ICU) transfer or death” and survival for each risk-category.

Risk Category	Score Range	Observed 20-Day Event-Free Survival ^1^	Observed 20-Day Survival ^2^
“Low risk”	score ≤ 15	0.97 (95% CI, 0.87 to 0.99)	0.98 (95% CI, 0.87 to 0.99)
“Intermediate risk”	15 < score < 40	0.67 (95% CI, 0.51 to 0.79)	0.76 (95% CI, 0.58 to 0.87)
“High risk”	score ≥ 40	0.24 (95% CI, 0.10 to 0.40)	0.49 (95% CI, 0.28 to 0.66)

^1^ Defined as “1–cumulative probability of ICU transfer or death.” ^2^ Defined as “1–cumulative probability of death.”

## Supplementary Materials

The following are available online at https://www.mdpi.com/2077-0383/9/5/1548/s1, Figure S1: Predictors of clinical deterioration, leading to ICU transfer or death, in hospitalized COVID-19 patients: univariable Kaplan–Meier survival curves, Figure S2: Clinical prediction index scoring algorithm, Figure S3: Calibration (Kaplan–Meier) curves showing optimal agreement between the prediction established by the prognostic score and the actual observation across the three risk-categories (i.e., the “low risk” category (upper curve), the “intermediate risk” (intermediate curve), and the “high risk” category (lower curve)), Table S1: Humanitas Covid-19 task force.
