# Supplementary material for: Early Predictors of Clinical Deterioration in a Cohort of 239 Patients Hospitalized for Covid-19 Infection in Lombardy, Italy
Source: J Clin Med. 2020 May 20;9(5):1548. doi: 10.3390/jcm9051548 (PMC7290833; doi:10.3390/jcm9051548)
Supplement: Supplementary file 1 [file jcm-09-01548-s001.zip › Supplementary Appendix 1 --- Figure S1.doc]

**Figure S1.** Predictors of clinical deterioration, leading to ICU transfer or death, in hospitalized COVID-19 patients: univariable Kaplan–Meier survival curves.

# Demographic characteristics

**Clinical characteristics**

**Signs and symptoms at admission**

# Comorbid conditions

Imaging features

**Laboratory characteristics**

**Blood routine**

**Infection related biomarkers**

**Blood biochemistry**

Coagulation function and other biomarkers
