# Supplementary material for: Early Predictors of Clinical Deterioration in a Cohort of 239 Patients Hospitalized for Covid-19 Infection in Lombardy, Italy
Source: J Clin Med. 2020 May 20;9(5):1548. doi: 10.3390/jcm9051548 (PMC7290833; doi:10.3390/jcm9051548)
Supplement: Supplementary file 1 [file jcm-09-01548-s001.zip › Supplementary Appendix 2 --- Figure S2.doc]

**Figure S2.** Clinical prediction index scoring algorithm.

1. **C-reactive protein serum levels**
a. Obtain the patient’s C-reactive protein (CRP) serum levels.
b. Assign as many points as mg/dL of CRP (e.g. if CRP = 7.73 then assign 7.73 points).
2. **Medical history of coronary heart disease**
a. Assign 10 points if the patient has coronary heart disease, 0 if not.
3. **Serum creatinine levels**
a. Assign the score associated with the patient’s creatinine levels (mg/dL) from the look up table.

| Creatinine look up table | |
| --- | --- |
| ***Creatinine (mg/dL)*** | ***Score*** |
| < 1.10 | 0 |
| ≥ 1.10 | 10 |

4. **Ratio of PaO_2_ to FiO_2_**
a. Assign the score associated with the patient ratio of PaO_2_ to FiO_2_ from the look up table

| Ratio of PaO_2_ to FiO_2_ look up table | |
| --- | --- |
| ***Ratio of PaO_2_ to FiO_2_*** | ***Score*** |
| PaO_2_/FiO_2_ > 300 | 0 |
| 200 < PaO_2_/FiO_2_ ≤ 300 | 10 |
| 100 < PaO_2_/FiO_2_ ≤ 200 | 25 |
| 100 ≤ PaO_2_/FiO_2_ | 30 |

5. **Respiratory rate (breaths per minute)**
a. Obtain the patient’s respiratory rate.
b. Assign 10 points if the patient’s respiratory rate is ≥ 20 breaths per minute, 0 if not.
6. **Overall Score**
a. Calculate 1b + 2a + 3a +4a + 5b =
b. Categorize the patient into *“low”*, *“intermediate”* or *“high risk”* of clinical deterioration

within 20 days by using the score obtained in 6a and the categorization table.

| Categorization table | |
| --- | --- |
| ***Category*** | ***Cut-offs*** |
| “Low risk” | score ≤ 15 |
| “Intermediate risk” | 15 < score < 40 |
| “High risk” | score ≥ 40 |
