# Supplementary material for: Early Predictors of Clinical Deterioration in a Cohort of 239 Patients Hospitalized for Covid-19 Infection in Lombardy, Italy
Source: J Clin Med. 2020 May 20;9(5):1548. doi: 10.3390/jcm9051548 (PMC7290833; doi:10.3390/jcm9051548)
Supplement: Supplementary file 1 [file jcm-09-01548-s001.zip › Supplementary Appendix 3 --- Figure S3.doc]

**Figure S3.** Calibration (Kaplan–Meier) curves showing optimal agreement between the prediction established by the prognostic score and the actual observation across the three risk-categories (ie, the *“low risk”* category [upper curve], the *“intermediate risk”* [intermediate curve], and the *“high risk”* category [lower curve]).
