# Supplementary material for: Early Predictors of Clinical Deterioration in a Cohort of 239 Patients Hospitalized for Covid-19 Infection in Lombardy, Italy
Source: J Clin Med. 2020 May 20;9(5):1548. doi: 10.3390/jcm9051548 (PMC7290833; doi:10.3390/jcm9051548)
Supplement: Supplementary file 1 [file jcm-09-01548-s001.zip › Supplementary Appendix 4 --- Humanitas Covid-19 Task Force.doc]

| Humanitas Covid-19 Task Force | |
| --- | --- |
|  | |
| ACCORNERO | STEFANO |
| Aghemo | Alessio |
| ALI | HUSSAM |
| ANGELINI | CLAUDIO |
| ARCARI | IVAN |
| AROSIO | PAOLA |
| Azzolini | Elena |
| Baccarin | Alessandra |
| BADALAMENTI | SALVATORE |
| BAGGIO | SARA |
| BARBAGALLO | MICHELA |
| BARBERI | CATERINA |
| BARBIC | FRANCA |
| BARBIERI | VIVIANA |
| BARBONE | ALESSANDRO |
| BASCIU | ALESSIO |
| Bocciolone | Monica |
| BOREA | FEDERICA |
| BORRONI | MARIO |
| BRESCIANI | GIANLUIGI |
| Brunetta | enrico |
| BULLETTI | CINZIA |
| CADONATI | CRISTINA |
| CALABRO' | LOrENZO |
| CALATRONI | MARTA |
| CALVETTA | ALBANIA ANTONIETTA |
| CANNATA | Francesco |
| Canziani | Lorenzo |
| CAPRETTI | GIOVANNI LUIGI |
| CARLANI | ELISA |
| CARRONE | FLAMINIA |
| Casana | Maddalena |
| cecconi | maurizio |
| CERIOTTI | CARLO |
| Ciccarelli | michele |
| CIMINO | MATTEO |
| CIUFFINI | LEONARDO |
| COLAIZZI | CHIARA |
| COLAPIETRO | Francesca |
| COSTA | GUIDO |
| COZZI | OTTAVIA |
| CRAVIOTTO | VINCENZO |
| CRESPI | CHIARA |
| CRIPPA | MASSIMO |
| DA RIO | LEONARDO |
| DAL FARRA | SARA |
| D'ANTONIO | FEDERICA |
| DE AMBROGGI | GUIDO |
| DE DONATO | MASSIMO |
| DE LUCIA | FRANCESCA |
| De Santis | Maria |
| DELLE ROSE | GIACOMO |
| DI PILLA | MARINA |
| DIPAOLA | FRANCA |
| DIPASQUALE | ANDREA |
| DIPASQUALE | ANGELO |
| DROANDI | GINEVRA |
| FAZIO | ROBERTA |
| FERRANTE | GIUSEPPE |
| FERRARA | ELISA CHIARA |
| FERRARI | MATTEO CARLO |
| FERRI | SEBASTIAN |
| folci | marco |
| FORESTI | SARA |
| FRANCHI | ELOISA |
| FRAOLINI | ELIA |
| Fugazza | alessandro |
| FURFARO | FEDERICA |
| GALIMBERTI | PAOLA |
| Galtieri | Alessia Piera |
| GAVAZZI | FRANCESCA |
| GENERALI | ELENA |
| GOLETTI | BENEDETTA |
| Guidelli | Giacomo |
| JACOBS | FLAVIA |
| KURIHARA | HAYATO |
| Lagioia | Michele |
| LIBRE' | LUCA |
| Lleo | Ana |
| LOIACONO | FERDINANDO |
| LUGHEZZANI | GIOVANNI |
| MACCALLINI | MARTA |
| MAIORINO | ALFONSO FRANCESCO |
| Malesci | Alberto |
| MANTOVANI | RICCARDO |
| MARCHETTINI | DAVIDE |
| MARINELLO | ARIANNA |
| MARKOPOULOS | NIKOLAOS |
| MASETTI | CHIARA |
| MILANI | ANGELO |
| MIRANI | MARCO |
| Morelli | Paola |
| MOTTA | FRANCESCA |
| Mundula | Valeria |
| NIGRO | MATTIA |
| omodei | paolo |
| ORMAS | MONICA |
| PAGLIARO | ARIANNA |
| PALIOTTI | ROBERTA |
| Parigi | Tommaso Lorenzo |
| PEDALE | ROSA |
| PEGORARO | FRANCESCO |
| Pellegatta | Gaia |
| PELLEGRINO | MARTA |
| PETRIELLO | GENNARO |
| PICCINI | SARA |
| Pocaterra | Daria |
| POLIANI | LAURA |
| PREATONI | PAOLETTA |
| PROCOPIO | FABIO |
| PUGGIONI | FRANCESCA |
| PUGLIESE | LUCA |
| RACCA | FRANCESCA |
| RANDAZZO | MICHELE |
| REGAZZOLI LANCINI | DAMIANO |
| REGGIANI | FRANCESCO |
| RODOLFI | STEFANO |
| RUONGO | LIDIA |
| SACCO | CLARA |
| Sandri | Maria Teresa |
| SAVI | MARZIA |
| SCARFO' | ISIDE |
| SHIFFER | DANA |
| SICOLI | FEDERICO |
| SOLANO | SIMONE |
| SOLITANO | Virginia |
| STAINER | ANNA |
| STELLA | MATTEO CARLO |
| STRANGIO | GIUSEPPE |
| TAORMINA | ANTONIO |
| TESTONI | LUCIA |
| Tordato | Federica |
| TRABUCCO | ANGELA |
| ULIAN | LUISA |
| VALENTINO | ROSSELLA |
| VALERIANO | CHIARA |
| VENA | WALTER |
| VERLINGIERI | SIMONA |
| Vespa | Edoardo |
| Voza | antonio |
| ZANUSO | VALENTINA |
| ZILLI | ALESSANDRA |
